# Supplementary material for: Occurrence, Genetic Variability of Tomato Yellow Ring Orthotospovirus Population and the Development of Reverse Transcription Loop-Mediated Isothermal Amplification Assay for Its Rapid Detection
Source: Viruses. 2022 Jun 27;14(7):1405. doi: 10.3390/v14071405 (PMC9323093; doi:10.3390/v14071405)
Supplement: Supplementary file 1 [file viruses-14-01405-s001.zip › viruses-1740633-supplementary-Figure S1.pdf]

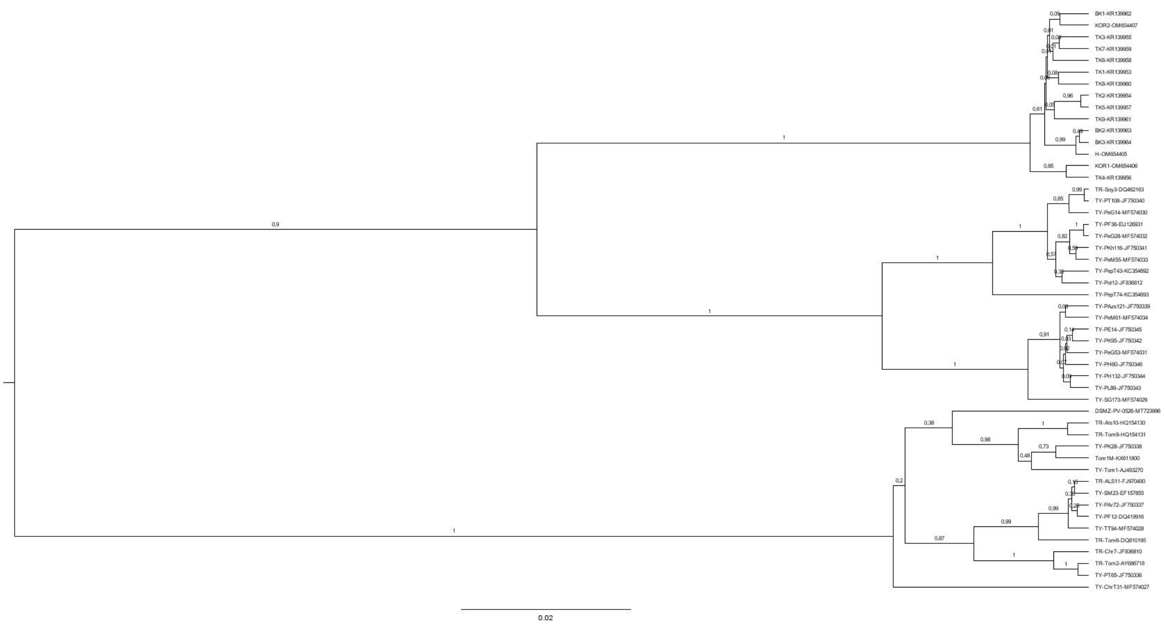

Figure S1. MCC tree constructed based on 50 TYRV full-length nucleocapsid (N). protein gene nucleotide sequences. Numbers above the brunches represent Bayesian posterior probabilities (BPP).
